# Propensity score-matched analysis of the ‘2+2’ parathyroid strategy in total thyroidectomy with central neck dissection

**DOI:** 10.3389/fendo.2025.1646573

**Published:** 2025-09-04

**Authors:** Hao Gong, Simei Yao, Tianyuchen Jiang, Yi Yang, Yuhan Jiang, Zhujuan Wu, Anping Su

**Affiliations:** ^1^ Division of Thyroid & Parathyroid Surgery, Department of General Surgery, West China Hospital Sichuan University, Chengdu, China; ^2^ Maxillofacial Surgery Department of Plastic Surgery Hospital, Chinese Academy of Medical Sciences and Peking Union Medical College, Beijing, China; ^3^ Lung Cancer Center/Lung Cancer Institute, West China Hospital Sichuan University, Chengdu, China

**Keywords:** parathyroid protection, propensity score matching, hypoparathyroidism, postoperative parathyroid function, parathyroid autotransplantation

## Abstract

**Objective:**

To evaluate the clinical efficacy of the “2+2” strategy (preserving 2 superior glands *in situ* and autotransplanting 2 inferior glands) in patients with papillary thyroid carcinoma (PTC) undergoing total thyroidectomy (TT) with bilateral central lymph node dissection (BCLND), using propensity score matching (PSM) to control confounding.

**Materials and methods:**

A retrospective cohort of 1,099 PTC patients treated with TT+BCLND at West China Hospital (2017–2023) was analyzed. After 1:1 PSM, 592 patients (296 per group) were included. Outcomes included temporary hypoparathyroidism (THP), permanent hypoparathyroidism (PHP), and postoperative PTH, calcium (Ca), and vitamin D (VitD) levels. Logistic regression identified predictors of THP and PHP.

**Results:**

After matching, baseline characteristics were comparable. The “2+2” group had longer operative time (150 vs. 123 min, *p*<0.01), higher THP incidence (72.97% vs. 48.31%, *p*<0.01), and lower PHP incidence (0.68% vs. 3.72%, *p* = 0.03). PTH and Ca levels dropped more on postoperative day 1 in the “2+2” group but recovered more rapidly between day 1 and month 1. By month 12, levels converged in both groups. Parathyroid autotransplantation was an independent risk factor for THP (OR = 2.476, *p*<0.01) but protective against PHP (OR = 0.139, *p* = 0.02). Tumor size was also associated with THP risk (OR = 1.424, *p* = 0.04).

**Conclusion:**

The “2+2” strategy increases short-term THP risk but significantly reduces long-term PHP. Rapid biochemical recovery supports the functional viability of autotransplanted glands. This approach may offer a safe and effective strategy for parathyroid management in high-risk thyroid surgeries.

## 1 Introduction

Thyroid cancer currently ranks as the ninth most common malignant tumor worldwide, with its occurrence rate showing a continuous increase over recent decades (1). In China, the age-standardized occurrence rate of thyroid cancer rose significantly from 0.85 per 100,000 in 1990 to 2.75 per 100,000 in 2019 (2). Papillary thyroid carcinoma (PTC) is the most prevalent pathological type of thyroid cancer (3). Total thyroidectomy (TT), as one of the profound therapeutic approaches for PTC, has significantly improved tumor prognosis. For patients with central lymph node metastasis or high recurrence risk, TT combined with bilateral central lymph neck dissection (BCLND) can further reduce recurrence risk (4). Although PTC has a favorable prognosis, long-term survival means that postoperative complications from surgery remain a major factor affecting patients’ quality of life (5, 6). In particular, postoperative hypoparathyroidism (HP) caused by surgical intervention has become a key challenge in the clinical management of PTC patients.

The pathological hallmark of HP is insufficient secretion of parathyroid hormone (PTH), leading to hypocalcemia and associated clinical symptoms (e.g., limb paresthesia, muscle cramps, and osteoporosis) (7). Based on disease duration, HP is classified into temporary hypoparathyroidism (THP) and permanent hypoparathyroidism (PHP), with reported complication rates varying substantially across institutions (8–14). While THP typically exerts transient and reversible effects on patients’ quality of life, PHP poses more profound consequences, particularly due to persistent hypocalcemic symptoms and skeletal/cardiovascular impairments (7).

In recent years, parathyroid autotransplantation (PA) has gained increasing attention as an intraoperative technique for preserving parathyroid function (15). The 2018 American Thyroid Association (ATA) guidelines recommend *in situ* preservation of all parathyroid glands during thyroidectomy, reserving PA only for cases with compromised vascular supply or accidental resection to mitigate postoperative HP risk (7). A retrospective study demonstrated that patients with all four parathyroid glands preserved *in situ* had a PHP incidence of 2.6%, significantly lower than those with only 1–2 glands preserved (16%) (16). Other studies corroborate that fewer in situ-preserved glands correlate with higher PHP rates, underscoring the priority of total *in situ* preservation (17). However, Sitges-Serra et al. (2018) noted that despite widespread recommendations, robust evidence supporting PA’s efficacy in PHP prevention remains limited (18). Conversely, multiple studies advocate PA’s effectiveness. Wei et al. (2014) reported a PHP incidence of 0.9% in the PA group versus 3.8% in the *in situ* preservation group among 477 patients subjected to TT+BCLND (10). Similarly, Zhang et al. (2022) observed PHP rates of 0.625% (PA) versus 5% (control) (19). Cheng et al. further confirmed these findings, noting significantly higher PTH levels in the PA group at 1, 3, and 6 months postoperatively (20). Wang et al. identified PA as a protective factor against PHP (OR: 0.27; 95% CI: 0.14–0.55, p<0.001) (21).

For high-risk patients undergoing TT+BCLND, we propose a “2+2” strategy: *in situ* preservation of bilateral superior parathyroid glands combined with intentional autotransplantation of bilateral inferior glands to reduce PHP incidence. Leveraging a large retrospective cohort and propensity score matching (PSM) to control confounders, we aim to precisely evaluate functional outcomes between this approach and complete *in situ* preservation. This study seeks to generate robust evidence to optimize parathyroid management in high-risk thyroid surgeries.

## 2 Methods

### 2.1 Study design and patient selection

This study retrospectively analyzed patients who underwent TT combined with BCLND for PTC at the Department of Thyroid Surgery, West China Hospital of Sichuan University, from December 2017 to November 2023. The study was approved by the Ethics Committee of West China Hospital, Sichuan University (Approval No. 20221525), and registered in the Chinese Clinical Trial Registry (Registration No. ChiCTR2200067079).

Inclusion criteria were: (1) histopathologically confirmed PTC without mixed histologic types; (2) initial surgical procedure including TT and BCLND, with or without lateral neck dissection; (3) detailed operative records indicating the number, location, and intraoperative handling (preservation vs. autotransplantation) of parathyroid glands; (4) no evidence of preoperative parathyroid disease or abnormal serum calcium (Ca) levels; (5) absence of prior neck surgery or radiotherapy; (6) complete follow-up data for ≥6 months, including PTH and Ca levels.

Exclusion criteria included: history of neck irradiation, previous thyroid/parathyroid surgery, concurrent malignancies, chronic organ dysfunction, pregnancy/lactation, incomplete TT+BCLND, or missing key biochemical or follow-up data.

Patients were retrospectively assigned to groups based on operative records. The intraoperative decision for autotransplantation versus *in situ* preservation was based on the surgeon’s objective assessment of parathyroid gland vascularity during surgery. Baseline clinical data, laboratory results, operative findings, and postoperative outcomes were extracted from electronic medical records and follow-up interviews.

### 2.2 Laboratory parameters and definitions

Laboratory reference ranges were based on standards from the Clinical Laboratory Department of West China Hospital: (1) PTH: 1.6–6.9 pmol/L; (2) Ca: 2.1–2.7 mmol/L; (3) Magnesium (Mg): 0.66–1.07 mmol/L; (4) Phosphorus (P): 0.81–1.45 mmol/L; (5) Vitamin D (VitD): ≥30 ng/mL; (6) Thyroglobulin (Tg): <77 ng/mL. THP was defined as a postoperative serum PTH level below the lower normal limit that returned to normal within 6 months (7). PHP was defined as persistently subnormal PTH levels beyond 6 months after surgery (7).

Recurrence was defined as either local recurrence or distant metastasis confirmed by cytology, histopathology, or radiologic imaging. Local recurrence referred to tumor regrowth in the thyroid bed or cervical lymph nodes after surgery, while distant metastasis indicated tumor spread to other tissues or organs following surgery (22).

### 2.3 Propensity score matching

Logistic regression was used to generate propensity scores based on the following covariates: age, sex, body mass index (BMI), tumor size, capsular invasion, T stage, N stage, scope of lymph node dissection, number of dissected and metastatic lymph nodes, and surgical duration. Patients were matched 1:1 using the nearest-neighbor method without replacement and a caliper width of 0.02 of the standard deviation of the logit of the propensity score. Balance between groups before and after matching was evaluated using standardized mean differences (SMD), with SMD <0.1 considered acceptable.

### 2.4 Statistical analysis

Continuous variables were expressed as mean ± standard deviation or median (interquartile range) and compared using Student’s *t*-test or Mann–Whitney *U* test. Categorical variables were compared using chi-square or Fisher’s exact test. A two-sided *p* value <0.05 was considered statistically significant. Statistical analysis was performed using SPSS Statistics version 29 and Python 3.10. Univariate analyses (chi-square test or Mann–Whitney U test) were used to explore associations between clinical variables and outcomes, including THP, PHP, and recurrence. Multivariate logistic regression was employed to identify independent predictors after adjusting for potential confounders. L2-regularization was applied to prevent model overfitting and improve stability.

## 3 Results

### 3.1 Baseline demographic and clinical characteristics after PSM

Among 1,099 PTC patients who TT+BCLND at West China Hospital from December 2017 to November 2023, 592 patients were successfully matched using propensity score matching (1:1 ratio), with 296 patients in each group. The distribution of surgical strategies remained relatively stable throughout the study period, with no significant temporal trend detected (chi-square test, p>0.05). Matching was based on variables related to THP, PHP, tumor recurrence/metastasis, and operative time to minimize confounding.

SMD for all covariates after matching were < 0.10, indicating good balance between the two groups (**Supplementary Figure S1**). Detailed baseline characteristics after PSM are shown in **Table 1**. There were no significant differences between groups in terms of age (p=0.92), sex (p=0.86), BMI (p=0.18), tumor location (p=0.05), extent of lymph node dissection (p=0.70), T stage (p=0.45), N stage (p=0.86), or other relevant clinical variables, confirming the effectiveness of the PSM process. Operative time was significantly longer in the “2+2” group than in the preservation group (150 min vs. 123 min, p<0.01). The prevalence of nodular goiter was also higher in the “2+2” group (50.00% vs. 40.88%, p=0.03). Notably, the incidence of THP was significantly higher in the “2+2” group (72.97% vs. 48.31%, p<0.01), whereas PHP occurred significantly less frequently (0.68% vs. 3.72%, p=0.03).

**Table 1:** Baseline demographic and clinical characteristics of patients after PSM.

Characteristics	“2+2” Group	Preservation Group	p-value
Total (n)	296(100.00%)	296(100.00%)	/
Age, years	42.00(33.00-49.00)	40.00(32.00-51.00)	0.92
Sex			
Male/Female, % Male	81/215(27.36%)	84/212(28.38%)	0.86
BMI(kg/m²)	23.08(21.23-25.36)	22.66(20.83-25.20)	0.18
Nodular goiter, n (%)	148(50.00%)	121(40.88%)	0.03*
Follow-up duration (days)	1531(898.5-1867.3)	1409(794.8-1859.8)	0.23
Operative time (min)	150.0(120.0-226.0)	123.0(98.8-185.3)	<0.01*
Tumor location, n (%)			
Unilateral lobe	179(60.47%)	153(51.69%)	0.05
Bilateral lobes	95(32.09%)	108(36.49%)	
Isthmus	22(7.43%)	35(11.82%)	
Extent of lymph node dissection, n (%)			
Bilateral central compartment	198(66.89%)	199(67.23%)	0.70
Bilateral central + unilateral lateral	85(29.05%)	81(27.36%)	
Bilateral central + bilateral lateral	12(4.05%)	16(5.41%)	
Number of metastatic lymph nodes	3.00(0.00-7.00)	2.00(0.00-7.00)	0.68
Number of dissected lymph nodes	15.00(7.00-28.00)	13.00(7.00-29.00)	0.76
Lymph node metastasis rate	0.20(0.00-0.38)	0.17(0.00-0.33)	0.52
Length of hospital stay (days)	7.00(6.00-9.00)	7.00(6.00-8.00)	0.01*
T stage, n (%)			
T1a	150(50.68%)	149(50.34%)	0.45
T1b	89(30.07%)	77(26.35%)	
T2	25(8.45%)	37(12.50%)	
T3a	1(0.34%)	3(1.01%)	
T3b	21(7.09%)	15(5.07%)	
T4a	9(3.04%)	12(4.05%)	
T4b	1(0.34%)	2(0.68%)	
N stage, n (%)			
0	89(30.07%)	88(29.73%)	0.86
1a	118(39.86%)	124(41.89%)	
1b	89(30.07%)	84(28.38%)	
M stage, n (%)			
0	294(99.32%)	291(98.31%)	0.45
1	2(0.68%)	5(1.69%)	
AJCC stage, n (%)			
I	279(94.26%)	272(91.89%)	0.33
II	17(5.74%)	24(8.11%)	
Maximum tumor diameter (cm)	1.00(0.70-1.50)	1.00(0.70-1.80)	
Capsular invasion			
Yes/No, % Yes	254/42(85.81%)	253/43(85.47%)	1.00
THP			
Yes/No, % Yes	216/80(72.97%)	143/153(48.31%)	<0.01*
PHP			
Yes/No, % Yes	2/294(0.68%)	11/285(3.72%)	0.03*
Tumor recurrence/metastasis			
Yes/No, % Yes	2/294(0.68%)	5/291(1.69%)	0.45
Postoperative complications			
Yes/No, % Yes	21/275(7.09%)	26/270(8.78%)	<0.01*
Radioiodine therapy			
Yes/No, % Yes	143/153(48.31%)	139/157(46.96%)	0.54

PSM, propensity score matching; BMI, body mass index; THP, temporary hypoparathyroidism; PHP, permanent hypoparathyroidism; AJCC, American Joint Committee on Cancer. *P* values marked with an asterisk (*) indicate statistical significance at *p*<0.05.

### 3.2 PTH, VitD, and Ca levels after PSM

In both groups, PTH levels declined significantly after surgery and gradually recovered over time. The most substantial drop occurred on postoperative day 1: in the preservation group, the median PTH level decreased from 5.03 to 1.66 pmol/L, while in the “2+2” group, it dropped from 5.03 to 0.95 pmol/L. PTH levels gradually increased thereafter, approaching preoperative levels by month 12 (see **Supplementary Table S1**). The intergroup difference was statistically significant on postoperative day 1 (p<0.01), but not at subsequent time points. VitD levels showed relatively mild fluctuations in both groups. Slight increases were observed at 1 month postoperatively in both the preservation and “2+2” groups. The change in VitD levels reached statistical significance in the preservation group (p=0.02), but not in the “2+2” group (p=0.29). Ca levels also demonstrated significant postoperative changes, persisting through month 12 (p<0.01). The largest intergroup difference occurred on postoperative day 1.

PTH recovery patterns are illustrated in **Supplementary Figure S2**. Briefly, on postoperative day 1, both groups experienced a marked drop, with a greater decrease in the ‘2+2’ group (median: 0.95 vs 1.66 pmol/L, p<0.01). However, the ‘2+2’ group exhibited faster recovery between day 1 and month 1, with both groups converging toward similar levels by month 12 (approximately 4.35 pmol/L). Calcium recovery followed a similar pattern (**Supplementary Figure S3**), with the ‘2+2’ group showing greater initial decline but faster subsequent recovery. The intergroup difference in Ca levels was statistically significant on day 1 (p<0.01) but diminished thereafter, indicating convergent recovery trajectories.

### 3.3 Univariate and multivariate logistic regression analysis of THP after PSM

Patients were divided into a THP group (n=359, 60.64%) and a non-THP group (n=233, 39.36%) based on the occurrence of THP during follow-up. Compared with the non-THP group, patients in the THP group had significantly longer hospital stays (7.00–9.00 vs. 7.00–8.00 days, p<0.01), longer operative times (148.00 vs. 124.00 minutes, p<0.01), and larger tumor diameters (1.10 vs. 1.00 cm, p=0.04) (**Table 2**). In addition, the THP group had a significantly higher rate of PA (60.17% vs. 34.33%, p<0.01), higher postoperative complication rates (10.03% vs. 4.72%, p=0.03), and higher incidence of PHP (3.62% vs. 0.00%, p=0.01).

**Table 2:** Comparison of baseline clinicopathological characteristics between THP and non-THP patients after PSM.

Characteristics	PSM-THP	PSM-Non-THP	p-value
Total (n)	359	233	/
Age, years	41.00 (32.00–50.00)	41.00 (33.00–49.00)	0.56
Sex			
Male/Female, % Male	94/265 (26.18%)	71/162 (30.47%)	0.30
BMI(kg/m²)	22.85 (20.84–25.38)	23.11 (21.28–25.18)	0.51
Nodular goiter, n (%)	169 (47.08%)	100 (42.92%)	0.36
Follow-up duration (days)	1420.00 (818.00–1865.50)	1541.00 (869.00–1847.00)	0.38
Operative time (min)	148.00 (115.00–218.25)	124.00 (100.00–181.50)	<0.01*
Parathyroid Autotransplantation			
Yes/No, % Yes	216 (60.17%)	80/153 (34.33%)	<0.01*
Tumor location, n (%)			
Unilateral lobe	198 (55.15%)	134 (57.51%)	0.67
Bilateral lobes	128 (35.65%)	75 (32.19%)	
Isthmus	33 (9.19%)	24 (10.30%)	
Extent of lymph node dissection, n (%)			
Bilateral central compartment	231 (64.35%)	166 (71.24%)	0.21
Bilateral central + unilateral lateral	109 (30.36%)	58 (24.89%)	
Bilateral central + bilateral lateral	19 (5.29%)	9 (3.86%)	
Number of metastatic lymph nodes	3.00 (0.00–8.00)	2.00 (0.00–6.00)	0.07
Number of dissected lymph nodes	15.00 (8.00–30.00)	12.00 (6.00–24.00)	<0.01*
Lymph node metastasis rate	0.19 (0.00–0.36)	0.17 (0.00–0.33)	0.53
Length of hospital stay (days)	7.00 (7.00–9.00)	7.00 (6.00–8.00)	<0.01*
T stage, n (%)			
T1a	167 (46.52%)	132 (56.65%)	0.15
T1b	103 (28.69%)	64 (27.47%)	
T2	42 (11.70%)	20 (8.58%)	
T3a	3 (0.84%)	1 (0.43%)	
T3b	28 (7.80%)	8 (3.43%)	
T4a	14 (3.90%)	7 (3.00%)	
T4b	2 (0.56%)	1 (0.43%)	
N stage, n (%)			
0	102 (28.41%)	75 (32.19%)	0.17
1a	142 (39.55%)	100 (42.92%)	
1b	115 (32.03%)	58 (24.89%)	
M stage, n (%)			
0	355 (98.89%)	230 (98.71%)	1.00
1	4 (1.11%)	3 (1.29%)	
AJCC stage, n (%)			
I	334 (93.04%)	217 (93.13%)	1.00
II	25 (6.96%)	16 (6.87%)	
Maximum tumor diameter (cm)	1.10 (0.70–1.70)	1.00 (0.60–1.50)	0.04*
Capsular invasion			
Yes/No, % Yes	306/53 (85.24%)	201/32 (86.27%)	0.82
PHP			
Yes/No, % Yes	13/346 (3.62%)	0/233(0.00%)	<0.01*
Tumor recurrence/metastasis			
Yes/No, % Yes	4/355 (1.11%)	3/230 (1.29%)	1.00
Postoperative complications			
Yes/No, % Yes	36/323 (10.03%)	11/222 (4.72%)	0.03*
Radioiodine therapy			
Yes/No, % Yes	176/183 (49.03%)	106/127 (45.49%)	0.45

PSM, propensity score matching; BMI, body mass index; THP, temporary hypoparathyroidism; PHP, permanent hypoparathyroidism; AJCC, American Joint Committee on Cancer. *P* values marked with an asterisk (*) indicate statistical significance at *p*<0.05.

No significant differences were observed between the two groups regarding baseline characteristics such as sex, age, BMI, follow-up duration, tumor recurrence/metastasis, radioactive iodine treatment, nodular goiter, capsular invasion, number of metastatic or dissected lymph nodes, lymph node metastasis rate, tumor location, extent of lymph node dissection, or TNM stage (p>0.05).

**Figure 1:**
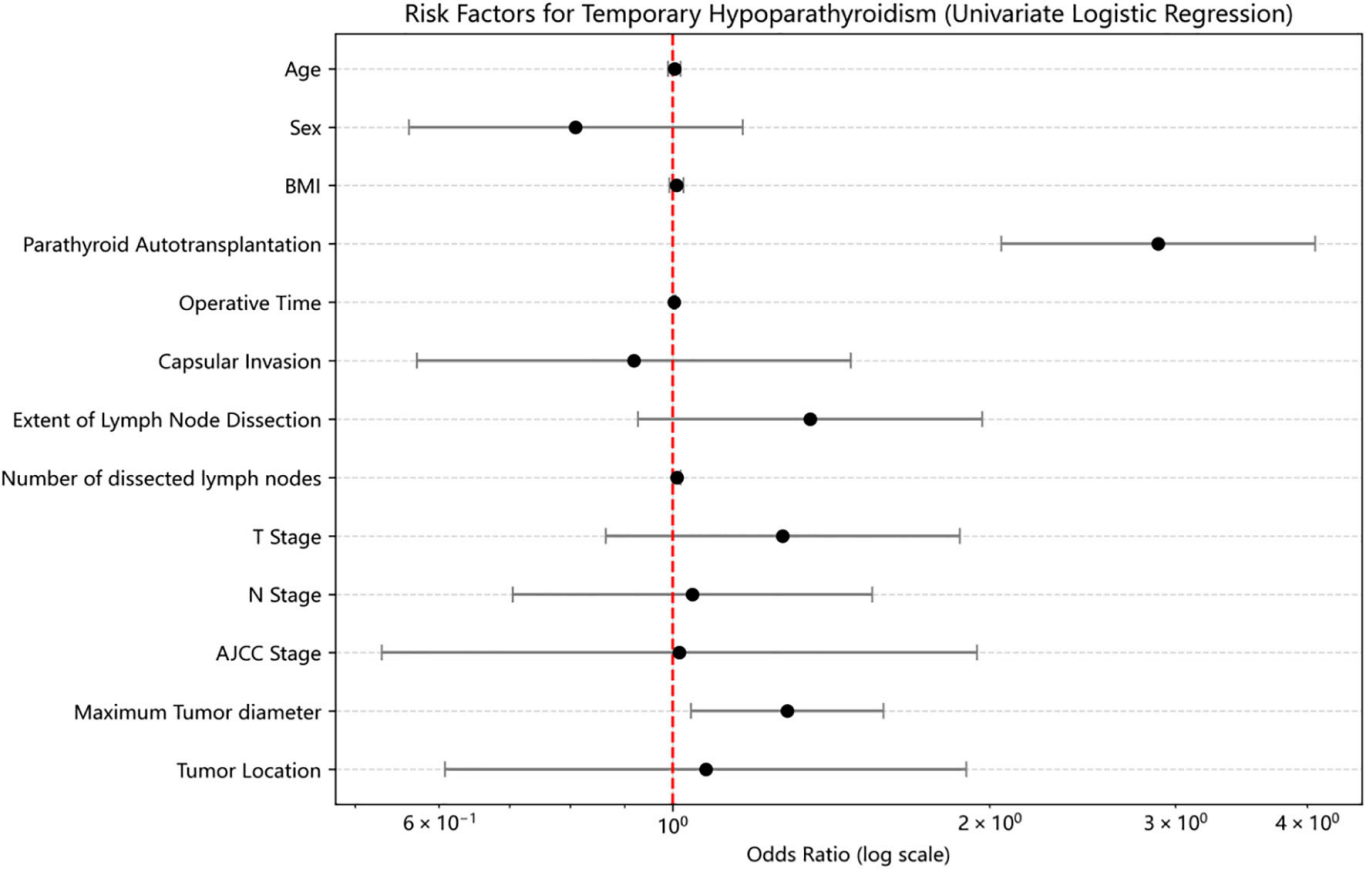
Risk factor analysis for THP after PSM.

Univariate logistic regression analysis revealed that PA was significantly associated with an increased risk of THP (OR = 2.889, 95% CI: 2.05–4.072, p<0.01) (**Figure 1**). Operative time was also a significant risk factor (OR = 1.003, 95% CI: 1.001–1.006, p<0.01). Other variables, including tumor location, sex, age, BMI, capsular invasion, tumor size, and number of dissected lymph nodes, were not significantly associated with THP (p>0.05).

In multivariate logistic regression analysis including age, sex, BMI, PA status, tumor location, operative time, capsular invasion, tumor size, number of dissected lymph nodes, extent of lymph node dissection, T stage, N stage, and AJCC stage, PA remained an independent risk factor for THP (OR = 2.476, 95% CI: 1.600–3.832, p<0.01). Additionally, tumor size was also independently associated with THP (OR = 1.424, 95% CI: 1.014–2.000, p=0.04).

### 3.4 Univariate and multivariate logistic regression analysis of PHP after PSM

Based on the occurrence of PHP, patients were divided into the PHP group (n=13, 2.20%) and the non-PHP group (n=579, 97.80%). Compared with the non-PHP group, patients in the PHP group had significantly higher THP incidence (100.00% vs. 59.76%, p=0.01), higher postoperative complication rates (38.46% vs. 7.25%, p<0.01), and lower rates of PA (15.38% vs. 49.22%, p=0.03) (**Table 3**). Additionally, the PHP group had a higher number of metastatic lymph nodes (8.00 vs. 2.00, p=0.02) and dissected lymph nodes (27.00 vs. 14.00, p=0.02). No statistically significant differences were observed between the two groups in terms of T stage, N stage, or AJCC stage. Other variables—including age, BMI, sex, operative time, intraoperative blood loss, length of hospital stay, follow-up duration, tumor size, radioactive iodine treatment, recurrence/metastasis, capsular invasion, tumor location, and comorbidities (e.g., Hashimoto’s thyroiditis, diabetes, hypertension)—showed no significant differences (p>0.05).

**Table 3:** Comparison of baseline clinicopathological characteristics between PHP and non-PHP patients after PSM.

Characteristics	PSM-PHP	PSM-Non-PHP	p-value
Total (n)	13	579	/
Age, years	51.00 (33.00–53.00)	41.00 (32.00–49.00)	0.24
Sex			
Male/Female, % Male	3/10 (23.08%)	162/417 (27.98%)	0.94
BMI(kg/m²)	23.05 (22.59–25.22)	22.91 (20.96–25.32)	0.29
Nodular goiter, n (%)	5 (38.46%)	264 (45.60%)	0.82
Follow-up duration (days)	1086.00 (765.00–1719.00)	1465.00 (841.00–1867.50)	0.29
Operative time (min)	195.00 (136.50–246.50)	140.00 (107.00–196.50)	0.16
Parathyroid Autotransplantation			
Yes/No, % Yes	2/11 (15.38%)	285/294 (49.22%)	0.03*
Tumor location, n (%)			
Unilateral lobe	7 (53.85%)	327 (56.48%)	0.32
Bilateral lobes	5 (38.46%)	196 (33.85%)	
Isthmus	1 (7.69%)	56 (9.67%)	
Extent of lymph node dissection, n (%)			
Bilateral central compartment	6 (46.15%)	391 (67.53%)	0.27
Bilateral central + unilateral lateral	6 (46.15%)	161 (27.81%)	
Bilateral central + bilateral lateral	1 (7.69%)	27 (4.66%)	
Number of metastatic lymph nodes	8.00 (2.00–14.00)	2.00 (0.00–7.00)	0.02*
Number of dissected lymph nodes	27.00 (14.00–33.00)	14.00 (7.00–28.00)	0.02*
Lymph node metastasis rate	0.27 (0.10–0.43)	0.18 (0.00–0.35)	0.18
Length of hospital stay (days)	8.00 (7.00–9.00)	7.00 (6.00–8.00)	0.13
T stage, n (%)			
T1a	7 (53.85%)	292 (50.43%)	0.73
T1b	2 (15.38%)	165 (28.50%)	
T2	2 (15.38%)	61 (10.54%)	
T3a	1 (7.69%)	34 (5.87%)	
T3b	1 (7.69%)	20 (3.45%)	
T4a	0(0.00%)	4 (0.69%)	
T4b	0(0.00%)	3 (0.52%)	
N stage, n (%)			
0	7 (53.85%)	237 (40.93%)	0.08
1a	5 (38.46%)	176 (30.40%)	
1b	1 (7.69%)	166 (28.67%)	
M stage, n (%)			
0	12 (92.31%)	573 (98.96%)	0.37
1	1 (7.69%)	6 (1.04%)	
AJCC stage, n (%)			
I	11 (84.62%)	540 (93.26%)	0.51
II	2 (15.38%)	39 (6.74%)	
Maximum tumor diameter (cm)	1.00 (0.70–1.80)	1.00 (0.70–1.60)	0.79
Capsular invasion			
Yes/No, % Yes	3/10 (23.08%)	82/497 (14.16%)	0.61
THP			
Yes/No, % Yes	13/0 (100.00%)	346/233 (59.76%)	<0.01*
Tumor recurrence/metastasis			
Yes/No, % Yes	1/12 (7.69%)	6/573 (1.04%)	0.37
Postoperative complications			
Yes/No, % Yes	5/8 (38.46%)	42/537 (7.25%)	<0.01*
Radioiodine therapy			
Yes/No, % Yes	3/10 (23.08%)	272/307 (46.98%)	0.06

PSM, propensity score matching; BMI, body mass index; THP, temporary hypoparathyroidism; PHP, permanent hypoparathyroidism; AJCC, American Joint Committee on Cancer. P values marked with an asterisk (*) indicate statistical significance at p<0.05.

**Figure 2:**
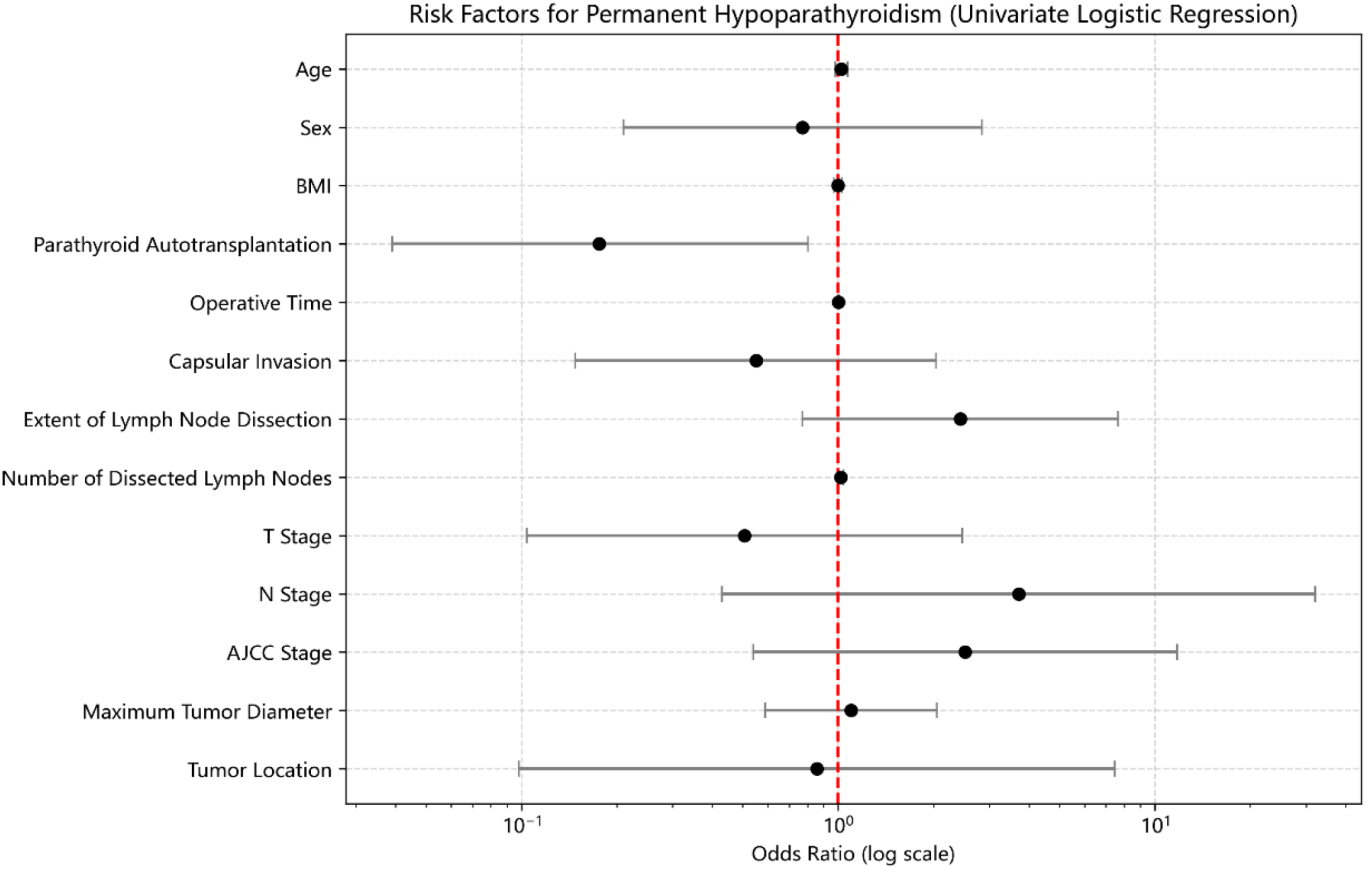
Risk factor analysis for PHP after PSM.

Univariate logistic regression analysis indicated that PA was significantly associated with a lower risk of developing PHP (OR = 0.176, 95% CI: 0.039–0.802, p=0.03) (**Figure 2**). No other variables—including age, sex, BMI, operative time, capsular invasion, tumor size, tumor location, number of dissected lymph nodes, extent of lymph node dissection, T stage, N stage, or AJCC stage—were significantly associated with PHP (p>0.05).

Multivariate logistic regression analysis incorporating age, sex, BMI, PA status, tumor location, operative time, capsular invasion, tumor size, number of dissected lymph nodes, extent of dissection, T stage, N stage, and AJCC stage confirmed that PA remained independently and negatively associated with the occurrence of PHP (OR = 0.139, 95% CI: 0.025–0.772, p=0.02). No other covariates reached statistical significance after adjustment.

## 4 Discussion

In this study, we systematically evaluated the impact of the “2+2” parathyroid protection strategy on postoperative parathyroid function in patients undergoing TT+BCLND using PSM. Our findings indicated that the “2+2” strategy was associated with a significantly higher incidence of THP but a substantially lower risk of PHP compared to complete *in situ* preservation. Additionally, patients in the “2+2” group exhibited greater initial declines in PTH and serum Ca levels postoperatively; however, faster recovery was observed thereafter, with no significant intergroup differences at 12 months. These observations suggest that despite a short-term suppression of parathyroid function, PA has the potential to recover and maintain stable function over the long term.

These findings align with previous reports demonstrating an initial postoperative period of functional quiescence for autotransplanted parathyroid glands, followed by gradual restoration of endocrine function due to revascularization and tissue repair. This study further supports this notion: on postoperative day 1, the “2+2” group exhibited a significantly greater decline in PTH levels; however, from day 1 to month 1, the rate of PTH recovery was markedly higher in the “2+2” group compared to the preservation group. This suggests that once the transplanted parathyroid tissue adapts to its new environment and re-establishes vascularization, its functional recovery may be more effective. Moreover, mild fluctuations in VitD levels in both groups indicated that PTH and Ca levels are the primary indicators reflecting postoperative parathyroid function.

Logistic regression analyses revealed that PA was independently associated with an increased risk of THP (OR = 2.476), yet served as a protective factor against PHP (OR = 0.139). This apparently contradictory finding underscores the intrinsic characteristics of PA—temporary functional suppression followed by a significant potential for long-term functional restoration. A plausible explanation is that glands preserved *in situ* with compromised blood supply might maintain short-term PTH secretion but are more likely to deteriorate in the long term. Conversely, transplanted glands, despite initial impairment, have considerable regenerative potential through subsequent revascularization.

Despite advances in refined anatomical techniques, it remains challenging to preserve the vascular supply to all parathyroid glands during surgery. A study involving 2,903 patients undergoing TT+BCLND reported that 64.84% of patients underwent *in situ* preservation of parathyroid glands, with an overall HP incidence of 21.94%, including 11.47% for THP and 10.47% for PHP (23). With the progressive development of surgical techniques and anatomical understanding, parathyroid protection strategies have evolved from basic approaches to more precise and individualized interventions (24, 25). We proposed the “2+2” protection strategy for TT+BCLND, in which inferior parathyroid glands are managed based on intraoperative vascular assessment. If the inferior glands are classified as B2 or B3—indicating favorable vascular anatomy—preservation *in situ* is preferred. Conversely, if blood supply is compromised, autotransplantation is recommended. The “2+2” strategy represents an innovative approach to parathyroid protection specifically tailored for TT+BCLND, aiming to maximize functional preservation. This strategy achieves dual optimization during surgery by balancing anatomical feasibility with functional outcomes.

However, not all patients undergoing TT+BCLND require PA. Excessive transplantation may lead to unnecessary medical costs, while insufficient transplantation may fail to effectively prevent PHP (26, 27). As such, developing risk stratification models to guide individualized intraoperative decision-making has become a key focus in current clinical research (20, 28). Studies have shown that patients with central lymph node metastasis often require more extensive tissue resection during surgery, thereby increasing the risk of parathyroid injury and the incidence of HP (29, 30). Therefore, patients staged as cN1 should be considered priority candidates for PA. In addition, patients with ectopic parathyroid glands located within the surgical field—especially when the ectopic glands are obscure or difficult to identify—are at higher risk of postoperative PHP due to the limited feasibility of effective *in situ* preservation, and may also benefit from PA (31–33). Similarly, patients undergoing reoperation typically face challenges such as tissue adhesions and distorted anatomy, making identification and preservation of parathyroid glands more difficult. PA should also be considered in these cases (34, 35). For patients with intraoperative evidence of parathyroid devascularization, timely PA has become a widely accepted practice and is recognized as a key measure for reducing the risk of PHP (4).

This study has several limitations. First, as a retrospective analysis, patient allocation was based on intraoperative findings rather than randomization, potentially introducing selection bias despite propensity score matching. Second, inter-surgeon variability in vascular assessment cannot be completely excluded. Third, the single-center design may limit generalizability. Fourth, we defined hypoparathyroidism based on PTH rather than calcium levels. While this provides a direct measure of parathyroid function less influenced by routine calcium supplementation, it may identify subclinical cases that would not meet calcium-based criteria, affecting comparability with other studies. Future prospective studies should validate different diagnostic criteria. Finally, longer follow-up would strengthen confirmation of the ‘2+2’ strategy’s long-term outcomes.

## 5 Conclusion

The “2+2” parathyroid protection strategy was associated with an increased short-term risk of THP but effectively reduced the incidence of PHP without adversely affecting mid- to long-term parathyroid function recovery. Therefore, the “2+2” strategy holds significant potential for functional protection in high-risk thyroid surgeries and warrants further validation and promotion through prospective clinical research.

## Data Availability

The raw data supporting the conclusions of this article will be made available by the authors, without undue reservation.
