# Supplementary material for: Propensity score-matched analysis of the ‘2+2’ parathyroid strategy in total thyroidectomy with central neck dissection
Source: Front Endocrinol (Lausanne). 2025 Sep 4;16:1646573. doi: 10.3389/fendo.2025.1646573 (PMC12443579; doi:10.3389/fendo.2025.1646573)
Supplement: Supplementary file 1 [file DataSheet1.docx]

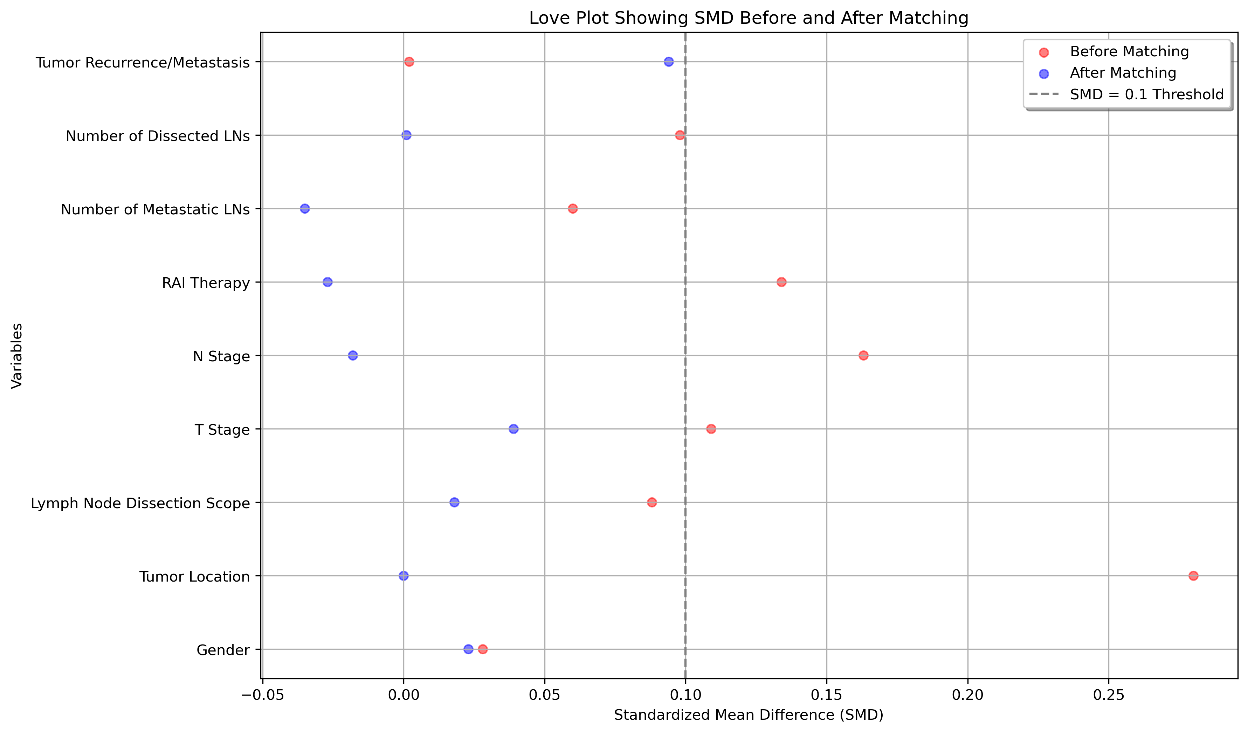


**Supplementary Figure S1. Assessment of Covariate Balance After PSM Using SMD**


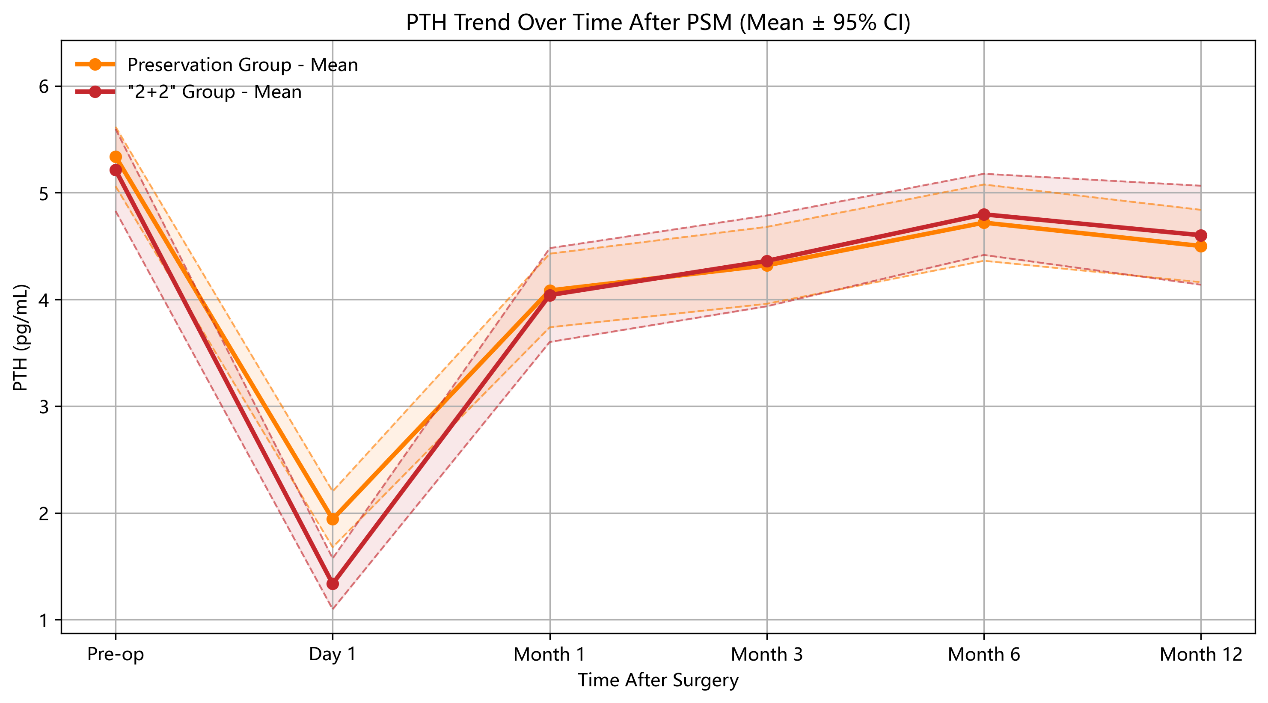


**Supplementary Figure S2. Time Trends of PTH Levels in Patients After PSM**


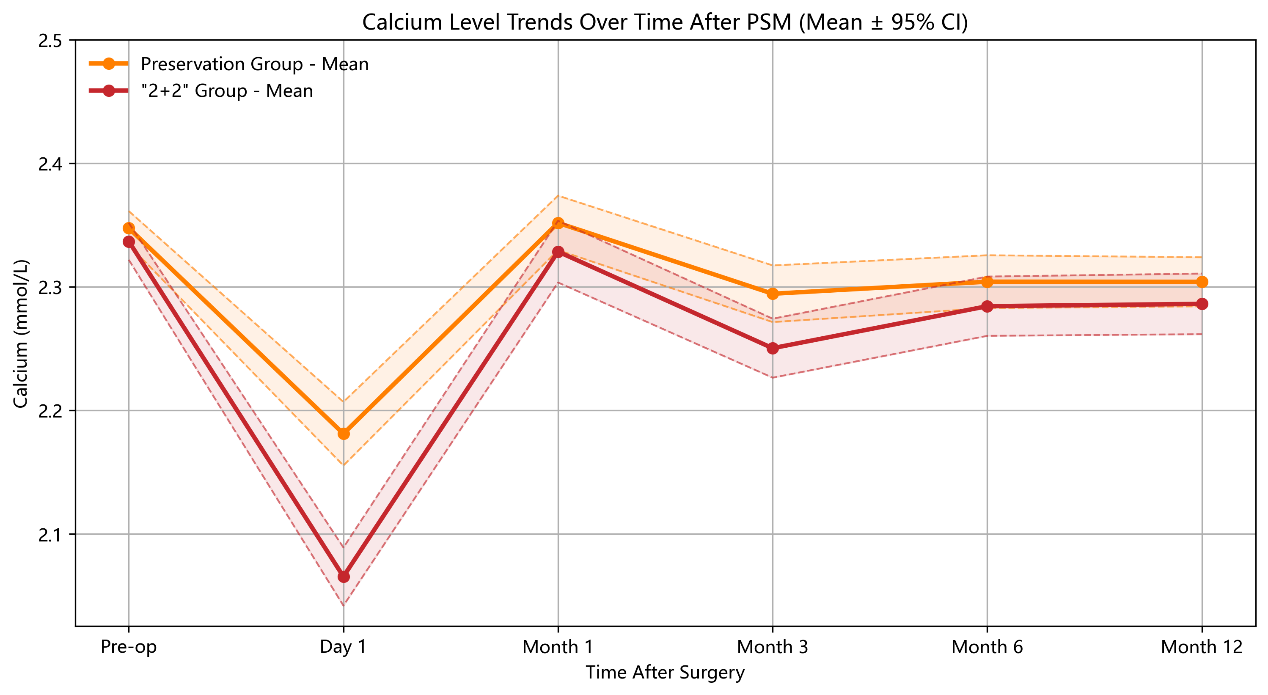


**Supplementary Figure S3. Time Trends of Ca Levels in Patients After PSM**

**Supplementary Table S1.** Serum PTH, Ca, VitD, and Tg levels in the preservation and “2+2” groups before and after surgery at each follow-up time point.

|  | Group | Pre-op | POD 1 | 1 mo | 3 mo | 6 mo | 12 mo | P1 value |
| --- | --- | --- | --- | --- | --- | --- | --- | --- |
| PTH(pmol/L) | 2+2 | 5.03(4.30-5.82) | 0.95(0.65-1.67) | 3.59(2.70-4.65) | 3.99(3.11-4.68) | 4.25(3.45-5.23) | 4.39(3.25-5.26) | ＜0.01* |
|  | Pres | 5.03(4.44-5.70) | 1.66(0.77-2.80) | 3.85(2.65-5.04) | 4.36(2.93-5.48) | 4.50(3.48-5.92) | 4.35(3.12-5.69) | ＜0.01* |
|  | P2 value | 0.796 | ＜0.01* | 0.230 | 0.155 | 0.165 | 0.899 | / |
| VitD(nmol/L) | 2+2 | 44.70(34.58-58.10) | 40.65(26.98-72.10) | 54.30(44.40-65.29) | 47.40(39.60-62.60) | 51.25(40.48-66.25) | 52.10(41.30-64.50) | 0.285 |
|  | Pres | 45.65(34.70-58.92) | 38.55(28.77-59.80) | 51.97(40.80-62.75) | 49.40(39.30-58.90) | 52.10(41.60-65.45) | 55.00(46.02-67.88) | 0.024 |
|  | P3 value | 0.738 | 0.847 | 0.048* | 0.973 | 0.957 | 0.084 | / |
| Ca(mmol/L) | 2+2 | 2.34(2.28-2.41) | 2.11(2.02-2.21) | 2.32(2.25-2.40) | 2.26(2.19-2.34) | 2.28(2.21-2.36) | 2.27(2.20-2.36) | ＜0.01* |
|  | Pres | 2.34(2.28-2.41) | 2.18(2.08-2.27) | 2.36(2.27-2.42) | 2.30(2.22-2.38) | 2.30(2.23-2.38) | 2.31(2.24-2.37) | ＜0.01* |
|  | P4 value | 0.831 | ＜0.01* | ＜0.01* | ＜0.01* | 0.038* | 0.010* | / |
| Tg(μg/L) | 2+2 | 14.50(3.48-29.10) | 9.72(1.55-31.57) | 0.04(0.04-0.30) | 0.08(0.04-0.74) | 0.04(0.04-0.24) | 0.04(0.04-0.10) | ＜0.01* |
|  | Pres | 19.09(6.41-53.00) | 7.84(0.74-260.08) | 0.10(0.04-0.41) | 0.12(0.04-1.62) | 0.04(0.04-0.23) | 0.04(0.04-0.16) | ＜0.01* |
|  | P5 value | ＜0.01* | 0.375 | 0.050* | 0.050* | 0.617 | 0.137 | / |

Group = Treatment group; Pre-op = Preoperative; POD 1 = Postoperative day 1; 1 mo / 3 mo / 6 mo / 12 mo = 1 / 3 / 6 / 12 months postoperatively; 2+2 = “2+2” parathyroid protection strategy group (bilateral superior glands preserved in situ, bilateral inferior glands autotransplanted); Pres = Complete in situ preservation group; P1 value = P value comparing groups at each time point from pre-op to POD1; P2 / P3 / P4 / P5 value = P values comparing the two groups at 1, 3, 6, and 12 months postoperatively, respectively.
